# Case Report: Safety and Efficacy of Denosumab in Four Children With Noonan Syndrome With Multiple Giant Cell Lesions of the Jaw

**DOI:** 10.3389/fped.2020.00515

**Published:** 2020-09-18

**Authors:** Kristen Ferriero, Biraj Shah, Yun Yan, Surya Khatri, John Caccamese, Joseph A. Napoli, Michael B. Bober, Janet L. Crane

**Affiliations:** ^1^Department of Pediatrics, Division of Genetics, Alfred I. duPont Hospital for Children, Wilmington, DE, United States; ^2^Department of Oral and Maxillofacial Surgery, John H. Jr, Stroger Hospital of Cook County, Chicago, IL, United States; ^3^Division of Endocrinology, Children Mercy Kansas City, University of Missouri- Kansas City, School of Medicine, Kansas City, MO, United States; ^4^Department of Pediatrics, Division of Endocrinology, Johns Hopkins University School of Medicine, Baltimore, MD, United States; ^5^Department of Oral and Maxillofacial Surgery, University of Maryland School of Dentistry, Baltimore, MD, United States; ^6^Division of Plastic and Reconstructive Surgery, Childrens Hospital of Philadelphia, Philadelphia, PA, United States; ^7^Department of Pediatrics, Division of Orthogenetics, Alfred I. duPont Hospital for Children, Wilmington, DE, United States

**Keywords:** Noonan syndrome, multiple giant cell lesions, denosumab, jaw, child

## Abstract

Noonan syndrome is a genetic disorder caused by mutations in the RAS/MAPK pathway. Multiple giant cell lesions are a rare sequelae of disruptions in this pathway, termed Noonan-like multiple giant cell lesions (NL/MGCLs). Medical management of these tumors rather than surgical intervention is preferential as the lesions are benign but locally destructive and recurring. This case series describes four male pediatric patients with Noonan syndrome and multiple giant cell lesions of the jaw treated with denosumab, a monoclonal antibody to receptor activator of nuclear factor kappa B ligand (RANKL), which has been approved for the treatment of malignant giant cell tumors in adults but not evaluated for safety or efficacy in children. All four pediatric patients responded clinically and radiographically to the treatment. Adverse events occurred in a predictable pattern and included hypocalcemia and joint pain during the initiation of treatment and symptomatic hypercalcemia after the cessation of treatment. Growth was not significantly impaired in these skeletally immature patients. This case series demonstrates how a weight-adjusted denosumab dose can effectively treat NL/MGCLs and provides laboratory data for consideration of the timing of monitoring for known side effects.

## Introduction

Noonan syndrome is a relatively common genetic syndrome occurring in an estimated 1 in 1,000–2,500 live births (1). Mutations in the Ras/mitogen-activated protein kinase (RAS/MAPK) pathway, which regulates cell growth, differentiation, senescence, and death, have been identified in Noonan syndrome. Multiple giant cell lesions (MGCLs), also known as central giant cell lesions or giant cell granulomas, are non-neoplastic lesions characterized by a proliferation of granulation tissue containing multinucleated giant cells embedded in a fibrous stroma (2). MGCL is a rare but typical complication of a dysregulated RAS/MAPK pathway, including Noonan syndrome, termed NL/MGCLS, and shares phenotypic characteristics of cherubism (3, 4). The underlying genetic mutations and sequelae distinguish the two. Mutations in *PTPN11* (5, 6) and *SOS1* (2, 7, 8) have been reported in people with NL/MGCLS, whereas mutations in *SH3BP2* are associated with cherubism (9). Giant cell lesions in cherubism tend to spontaneously resolve, whereas those observed in NL/MGCLS can have aggressive signs and symptoms (10–12). MGCLs most commonly occur in the jaw and joints (pigmented villonodular synovitis) (2).

MGCLs, as opposed to giant cell tumors of the bone, are benign but locally aggressive tumors that cause osteolytic destruction of bone (13). Multiple medical treatments have been explored with variable efficacy (11, 12). Surgical resection, similar to the tumor itself, often leads to disfigurement, especially in cases with jaw involvement. Enucleation and curettage has also been used but is associated with a high recurrence rate and does not completely avoid the morbidity associated with resection (14). An effective medical treatment option would be preferential. Both MGCLs and giant cell tumors are composed of osteoclast-like giant cells that express the receptor activator of nuclear factor kappa B (RANK) and mononuclear stromal cells that express RANK ligand (RANKL) (15), leading to osteoclast activation (16). Denosumab, a RANKL monoclonal antibody that inhibits osteolytic activation, is approved for malignant giant cell tumors in adults (16). Safety and efficacy of denosumab in MGCLs is limited to case reports, which have demonstrated successful avoidance of surgery and radiologic improvement with denosumab treatment. Only one case in an adult with Noonan syndrome has been published to date (17). Further data are needed, particularly in the pediatric population, to understand the efficacy and potential side effects on the growing skeleton.

## Case Description

**Figure 1:**
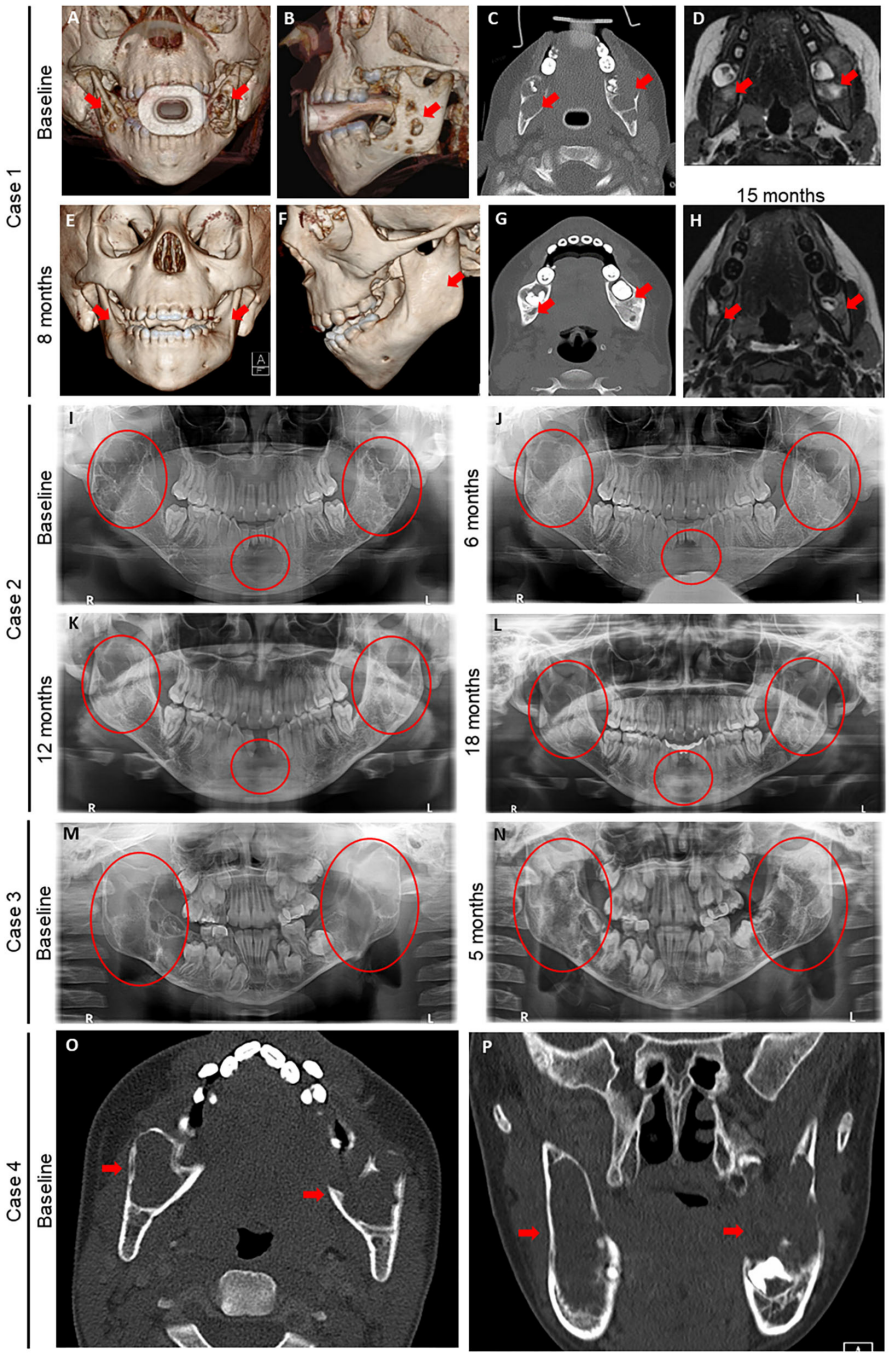
Radiography before and after denosumab in four children with Noonan-like/multiple giant cell lesions (NL/MGCLs). **(A–H)** Case 1 baseline images **(A–D)** of 3-dimensional (3D) computed tomography (CT) reconstruction of frontal **(A)** and profile **(B)** views, axial CT without contrast **(C)**, and axial magnetic resonance imaging (MRI) **(D)** at the level of mandibular molars relative to post-denosumab **(E–H)** 3D CT reconstruction of frontal **(E)** and profile **(F)** views, axial CT without contrast **(G)** 8 months after initial denosumab treatment, and axial magnetic resonance imaging (MRI) **(F)** at the level of mandibular molars 15 months after second denosumab treatment. **(I–L)** Case 2 dental orthopantomogram at baseline **(I)** and after on denosumab for 6 **(J)**, 12 **(K)**, and 18 **(L)** months. **(M, N)** Case 3: dental orthopantomogram at baseline **(M)** and after on denosumab for 5 months **(N)**. **(O,P)** Case 4: baseline axial **(O)** and coronal **(P)** CT without contrast at level of mandibular molars. Red arrows and circles denote NL/MGCLs.

**Figure 2:**
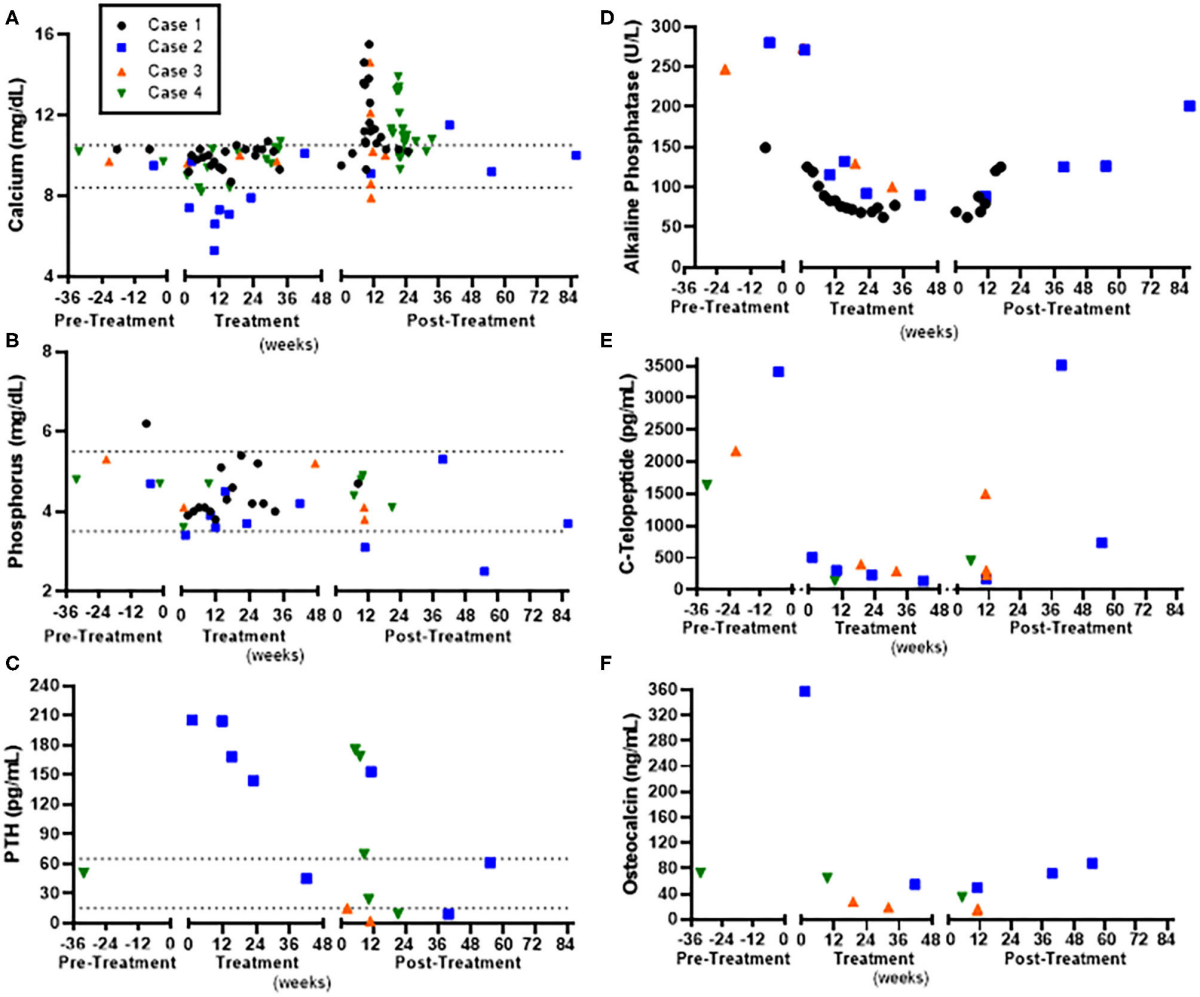
Laboratory assessment in relation to denosumab pre-treatment, during treatment, and post-treatment in four children with Noonan-like/multiple giant cell lesions (NL/MGCLs). **(A)** Serum calcium. **(B)** Serum phosphorus. **(C)** Parathyroid hormone (PTH). **(D)** Alkaline Phosphatase. **(E)** Serum carboxy-terminal telopeptide (C-Telopeptide), marker of bone resorption. **(F)** Osteocalcin, marker of bone formation. Normal ranges for calcium and phosphorus are indicated by dashed line.

This retrospective case series describes the safety and efficacy of denosumab treatment in four children (3, 17, 8, and 13 years old) with NL/MGCLs of the jaw. The diagnosis of Noonan syndrome was based on clinical phenotype in three of the patients, whereas one patient was recognized only after genetic testing for MGCLs. All patients had symptomatic lesions affecting tooth positioning, chewing, and/or facial disfigurement. Surgical and medical options were discussed, including surgical curettage, calcitonin, bisphosphonate, interferon, and denosumab. After discussions between the care team and families, the decision was made to try denosumab. Baseline labs and Dual-energy X-ray absorptiometry (DXA) were obtained. Weight-based dosing was calculated by using the approved adult dose (120 mg per month) and dividing by an average adult weight of 70 kg. Legal guardians of all patients signed written informed consent for inclusion in this report. Baseline characteristics of the four patients are shown in Table 1. NL/MGCLs improved grossly and radiographically within 6 months (Figure 1). Complications of treatment included joint pain, hypocalcemia, and hypercalcemia when initiating and discontinuing denosumab, respectively (Figure 2).

**Table 1:** Basic patient characteristics.

	**Case 1**	**Case 2**	**Case 3**	**Case 4**
Noonan mutation	*SOS1* heterozygous c.2536G>A (p.Glu846Lys), Exon 16, *SOS1*, pathogenic, *de novo*	ND	*PTPN11* gene, heterozygous, p.Asn200Tyr, c.598 A>T, likely pathogenic	*SOS1* heterozygous disease-associated missense mutation in exon 10 of the *SOS1* gene, c.1310T>C (p.Ile437Thr)
Age at Presentation	3 yo	17 yo	8 yo	13 yo
Physical features of Noonan syndrome	Ptosis, downslanting palpebral fissures, posteriorly angulated ears, cryptorchidism, and pectus excavatum	Downslanting palpebral fissures, rounded eyebrows, posterior rotation of ears with thickened helices, low hairline, wide neck, pulmonary stenosis and ASD, cryptorchidism, and short stature	Bilateral epicanthal folds, and slightly posteriorly rotated ears	Slightly increased inner canthal distance. Down slanting palpebral fissures. Rounded eyebrows. Posterior rotation of ears with thickened helices. Depressed nasal bridge. Slightly wide nasal tip. High, widely spaced peaks of the vermillion border. High palate. Low hairline. Upper pectus carinatum with lower pectus excavatum.
Laterality	Bilateral	Central	Bilateral	Bilateral
Number of Lesions	5	3	2	2
Size of Lesions	Left Mandible: 4.95 cm × 1.9 cm × 3.2 cm. Right Mandible: 4.0 cm × 1.42 cm × 2.6 cm. Right Maxilla: 2.6 cm × 1.7 cm × 1.5 cm. Left Maxilla: 1.65 cm × 2.15 cm × 0.98 cm. Anterior Left Maxilla: 1.1 cm × 1.1 cm × 2.2 cm	Anterior Mandible: 2.4 cm × 4.2 cm. Right Mandible: 5.0 cm × 4.3 cm. Left Mandible: 4.0 cm × 7.3 cm	Right Mandible: 3.6 cm × 6.0 cm × 4.2 cm. Left Mandible: 2.6 cm × 3.4 cm × 3.9 cm	Right Mandible: 2.5 cm × 1.5 cm × 5.6 cm SI. Left Mandible: 2.3 cm × 1.6 cm × 3.6

### Case 1

A 3-year-old male with Noonan syndrome (*SOS1* mutation) presented with asymptomatic jaw swelling and was found to have NL/MGCLs of the jaw bilaterally (Figure 1). He was started on denosumab 25 mg subcutaneously every 4 weeks. During treatment, calcium levels remained within normal limits without need for calcium or vitamin D supplementation. Response to treatment was monitored by MRI and physical exam. After four doses, the NL/MGCLs had significantly decreased in size. He received four additional monthly treatments. Denosumab was discontinued as his MRI showed stabilization of the lesions (Figure 1) and his bone mineral density (BMD) had increased (Table 2). About 2 months after discontinuation of denosumab, he refused to walk and was found to be hypercalcemic to 13.6 mg/dL. He was treated with hyperhydration, furosemide, calcitonin, and ultimately pamidronate (0.5 mg/kg/dose). X-rays of long bones showed dense bands in metaphyses consistent with anti-resorptive use (Supplemental Figure 1). He was able to bear weight as his calcium normalized. He was re-hospitalized for hypercalcemia (peak calcium 15.5 mg/dL) another 2 weeks later. Treatment included hyperhydration, calcitonin, and pamidronate, which decreased the serum calcium significantly prior to discharge. He had stable disease until about 2 years off treatment, when an MRI showed an enlargement of his NL/MGCLs, although not as large as in the initial presentation. Although his BMD had increased above the normal range after 8 months of denosumab, DXA prior to the start of the second treatment showed normalization of BMD (Table 2 and Supplemental Table 1). During this second round of denosumab, he was started on calcium supplementation (500 mg twice daily) due to reported poor dietary calcium intake. Denosumab dose was adjusted for weight gain to a maximum dose of 36 mg. Calcium and phosphorus levels remained within normal limits during treatment (Figure 2). Follow-up MRIs obtained 6 and 12 months after the re-initiation of treatment showed interval decrease in size and remineralization of the lesions. Denosumab was discontinued after 15 months as MRI showed stabilization of the lesions without continued improvement (Figure 1). He developed rebound hypercalcemia (peak calcium 15 mg/dL) requiring hospitalization and treatment with hyperhydration and pamidronate almost 4 months later.

**Table 2:** Treatment details.

	**Case 1**	**Case 2**	**Case 3**	**Case 4**
Dose	1.7 mg/kg (25–36 mg)	1.3–1.7 mg/kg (60 mg)	1.5–1.7 mg/kg (35 mg)	1.7 mg/kg (60 mg)
Treatment Frequency	Monthly	0, 7, 14, and 28 days for initiation, then every 4 weeks × 1 year, then every 3–4 months × 1 year	Monthly	Monthly
Treatment Duration	4/2015–12/2015. 4/2018–8/2019	07/2016–08/2018	05/2017–01/2018	09/2018–03/2019
Height *z*-score Pre-Treatment	−2.38; −2.64	−2.2	+0.1	−1.68
Height *z*-score Post-Treatment	−2.19; −3.01	−1.2	0.0	−1.44
Baseline Bone Mineral Density Height-adjusted *Z*-Scores	4/2015: Spine +0.26; 4/2018: Spine +0.86	8/2016: Spine −2.5; Total Body −3.4	1/2017: Spine −1.7; TBLH −1.3	8/2018: Spine +0.5
Bone Mineral Density Height-adjusted *Z*-Scores Post-Treatment	12/2015: Spine +2.8; 5/2019: Spine +2.9	2/2017: Spine −1.6; Total Body −2.6; TBLH −1.3	2/2018: Spine: +0.3; TBLH +1.0	3/2019: Spine +2.2
Notable Labs	Hypercalcemia (2/2016–3/2016 and 11/2019-12/2019)	Hypocalcemia (11/2016), hypercalcemia (5/2018)	Hypercalcemia (3/2018)	Hypocalcemia (12/2018), Hypercalcemia (7/2019)
Adverse Events	Hospital admission × 4 for pamidronate	No hospitalizations	Hospital admission × 1 for zoledronate	Knee pain during treatment course. Hands and toe aching in addition to worsened knee pain with difficulty walking 6 weeks after the injection was discontinued

### Case 2

A 17-year-old male with Noonan syndrome (diagnosed clinically in infancy) presented with recurrent NL/MGCLs of the jaw, status-post two surgical resections, 3 and 2 years prior. He had two benign lesions in the mandibular rami and one aggressive lesion in the symphysis. He reported alterations with eating secondary to loose teeth. After the correction of vitamin D deficiency, he was started on denosumab 60 mg, given at 0, 7, 14, and 28 days, then every 4 weeks for 1 year. He was advised to also take elemental calcium 500 mg three times daily. Dental orthopantomogram X-rays were taken approximately every 6 months (Figure 1). X-rays showed increasing radiopacity of the ramus lesions in response to denosumab, suggestive of increased calcification, and formation of normal bony trabeculation. Treatment did not prevent the loss of the two mandibular central incisors, but his other teeth solidified and he was able to eat food without pain. The bone resorption marker, carboxy-terminal collagen telopeptide (CTX), was elevated pre-treatment and suppressed to the normal range. The bone formation marker osteocalcin showed a similar pattern, elevated early in treatment that suppressed to normal range throughout the treatment phase (Figure 2). By 2 months of treatment, he developed symptomatic hypocalcemia (5.3 mg/dL). Parathyroid hormone levels were appropriately elevated. Calcium improved initially with increasing calcium and cholecalciferol supplementation. Calcitriol (0.25 mg daily) was started with recurrent hypocalcemia with waning supplementation compliance, which stabilized calcium. After 1 year of monthly denosumab, frequency was extended to every 3 months. Delay beyond 3 months resulted in symptomatic hypercalcemia (peak 11.5 mg/dL) and coincided with elevations in CTX, indicative of rebound hypercalcemia secondary to bone resorption. Symptoms, including unbearable knee and shoulder pain and mild elbow pain, resolved within 3 days after denosumab. To stop denosumab while reducing the risk of rebound hypercalcemia, he was started on alendronate (35 mg by mouth weekly for 1 month) 2.5 months after last denosumab injection. Calcium remained normal 24 weeks after the last denosumab injection. Of note, he was treated with growth hormone for short stature prior to and continued (17–19 years of age) while on denosumab. His height increased from a *z*-score of −2.15 to a *z*-score of −1.2 (Table 2). Bone age X-rays showed sclerotic bands in metaphyses (Supplemental Figure 1).

### Case 3

An 8-year-old male presented for a concern of mandibular MGCLs, initially noted at 5 years of age and monitored conservatively. By 8 years of age, he was noted to have facial disfigurement, with an expansion of two benign lesions of the mandible bilaterally, giving rise to a cherub-appearing face. He had mild pain associated with the contour and bulkiness of the lesions, which made chewing uncomfortable. Genetic workup revealed a *PTPN11* mutation consistent with Noonan syndrome. He was started on denosumab 35 mg subcutaneously every 4 weeks. Baseline labs and DXA were obtained (Figure 2 and Table 2). Cholecalciferol was prescribed to optimize 25-OH vitamin D. He received six denosumab doses over the course of 7.5 months. There was an unintentional gap of 2.5 months between the third and fourth dose. Serum calcium 4 days after initial denosumab dose and prior to the fourth dose was normal (Figure 2). CTX was elevated pre-treatment and suppressed to the normal range, while osteocalcin was suppressed below normal throughout the treatment phase. Within 5 months, he reported improvement in pain, including while eating. On physical exam, the mandible shape had normalized. Dental orthopantomogram X-rays showed improvement in radiodensity, suggesting improved calcification and bony trabeculation (Figure 2). There was also improvement in the path of eruption of the mandibular right first molar and left second molar, suggestive of resolving mandibular lesions. BMD by DXA increased but remained within the normal range for age. Densoumab frequency was extended. Two months between doses, calcium remained normal. However, further extension between doses resulted in symptomatic hypercalcemia (peak calcium 14.6 mg/dL) with arm and leg pain and abnormal gait after 10 weeks. X-rays of the extremities were normal, with the exception of a subcortical lucency of the distal metaphyses (Supplemental Figure 1). Serum CTX was elevated, indicative of increased bone resorption. He was given hyperhydration and zoledronate (0.0125 mg/kg). Calcium reached a nadir (7.9 mg/dL) ~48 h after zoledronate. Thereafter, calcium remained normal. Twenty-one months after the last denosumab, he denied any jaw pain or problems chewing but acknowledged intermittent leg pain associated with exercise. Physical exam showed normal jaw contour. Long bone x-rays remained normal (Supplemental Figure 1).

### Case 4

A 13-year-old male with Noonan syndrome (*SOS1* mutation) presented with expanding mandibular NL/MGCLs, noted 4 years prior, but were now causing jaw pain and physical deformity with a cherub-appearing face. CT scan revealed bilateral mandibular, expansile cysts with cortical thinning (Figure 1). No disruption to teeth was noted, but he had required two dental procedures for tooth extractions. He was started on denosumab 60 mg monthly. After 6 months, physical exam revealed decreased jaw asymmetry and facial deformity. Radiographically, there was evidence of the consolidation of the cystic masses. Complications during his treatment course included throbbing pain in his knees 1 week after denosumab. Knee pain became less severe with subsequent injections. Calcium levels were slightly low (8.4 mg/dL); therefore, he was advised to take calcium and vitamin D3 supplements (Figure 2). After 6 months of treatment, BMD by DXA increased above the normal range in the spine (Table 2) but remained within the normal range for age at the distal femur (Supplemental Table 1). Therefore, the denosumab frequency was extended. At 6 weeks, 25-OH vitamin D was slightly low (19 ng/mL) with elevated PTH (175 pg/mL); calcium was normal (9.8 mg/dL). He was restarted on vitamin D3 and calcium supplements, and then discontinued, when at 10 weeks post-treatment, calcium was in the upper end of normal (10.3 mg/dL). He reported difficulty walking secondary to pain all over his body, but worse in hands, toes, and knees. By 12 weeks, pain persisted, and calcium had increased to 10.7 mg/dL with elevated urine calcium to creatinine ratio (580 mg Ca/g Cr). He was given one additional dose of denosumab 30 mg, which provided some pain relief and improvement in urinary calcium to creatinine ratio (20 mg Ca/g Cr). Eight weeks later, calcium was again elevated (11.3 mg/dL), and he was started on alendronate (35 mg by mouth weekly, increased to 70 mg when calcium did not decrease). Pain significantly worsened with a peak calcium of 13.2 mg/dL requiring hospitalization and treatment with hyperhydration and zoledronate (0.0125 mg/kg). Calcium nadir (8.9 mg/dL) occurred ~36 h after zoledronate. Two weeks later, hypercalcemia returned and was successfully managed with oral alendronate (70 mg by mouth 1 weekly for 2 months). Knee and bone age X-rays performed throughout treatment were remarkable for dense sclerotic metaphyseal bands and widening of the growth plates (Supplemental Figure 1).

## Discussion

There is significant phenotypic heterogeneity in Noonan syndrome among our patients. Cardinal features include short stature, cardiovascular defects, broad or webbed neck, pectus deformity, developmental delay, cryptorchidism, and characteristic facies (18). In our cohort, three of the four patients had an established diagnosis of Noonan's syndrome prior to development of MGCLs, whereas the Noonan phenotype was not overtly obvious and diagnosis was not made until presentation with MGCLs in one of the four. Although rare, giant cell tumors of the jaw, which can arise from dysregulation of RAS/MAPK pathways, should also be considered part of the phenotypic presentation of Noonan syndrome (7). Given the differing clinical courses and potential additional systems that may be affected, genetic testing should be considered in children presenting with giant cell tumors of the jaw with specific testing for mutations in *PTPN11, SOS1*, and *SH3BP2* (2, 5–9).

Giant cell tumors are composed of osteoclast-like giant cells that have formed secondary to stromal cell expression of RANKL. Denosumab, a monoclonal antibody against RANKL, has been approved by the United States Food and Drug Administration and by the European Medicines Agency following a clinical study evaluating the efficacy for malignant giant cell tumors in skeletally mature adults and adolescents (19). Given the unknown and unstudied effects of denosumab on growing bones, the use of denosumab in children has been limited. To date, we have found reports of 17 children and adolescents in the literature who have been treated with denosumab for various indications, including osteogenesis imperfecta type VI (20), juvenile Paget's disease (21), giant cell tumors (22–26), fibrous dysplasia (27), osteoglyphonic dysplasia (28), cherubism (17), and non-syndromic giant cell lesions of the jaw (17, 26). The use of denosumab in NL/MGCL has been reported in one adult (17). The safety and efficacy in non-skeletally mature people with NL/MGCL have not previously been reported.

All of our patients showed resolution of symptoms associated with their mandibular NL/MGCLs, likely because of the regression of MGCLs within the mandible. In all cases, the MRI, CT scans, and dental orthopantograms showed regression of the MGCLs and improvement in the radiographic appearance of mandibular bone. As shown in Figure 2, there was a significant increase in the radiodensity of the mandibular bone. The improved radiographic appearance of these lesions is suggestive of normalization of mandibular bony architecture; however, this cannot be concluded without a biopsy of the newly formed bone within the previous lesions. We achieved very similar results with denosumab in our patients with NL/MGCLs relative to patients with non-syndromic central giant cell lesions (17, 26). Using denosumab, we were able to avoid drastic surgical interventions, which risk extensive facial disfigurement, damage to developing teeth, injury to inferior alveolar nerve, and altered growth of the mandible. Although imaging studies showed improvement of the bone density in the lesions, the bony expansion caused by these lesions did not reduce. Some patients may require recontouring of the facial bone to provide improved esthetics.

Observed side effects were similar as previously described, including hypocalcemia during treatment and rebound hypercalcemia with discontinuation (17, 20–27). Namely, three of the four patients experienced hypocalcemia with the initiation of denosumab, noted within 1 week after medication administration, which was corrected with calcium supplementation. One patient who had non-adherence to calcium supplementation had more severe protracted hypocalcemia, which improved with the addition of calcitriol. None required hospitalization for hypocalcemia. All four cases experienced rebound hypercalcemia when discontinuing denosumab. The timing of hypercalcemia occurred between 67 and 80 days after the last monthly denosumab dose (Figure 2A). Two of four patients were treated with additional doses of denosumab, which quickly resolved the hypercalcemia, but recurred again 57–90 days later. All four patients required treatment with a bisphosphonate. Three had significant enough hypercalcemia to warrant hospitalization and treatment with bisphosphonate infusion, whereas hypercalcemia was prevented in one case by using alendronate orally preemptively beginning about 50 days after the last denosumab dose and continuing for 1 full month. One case had further hypercalcemia after zoledronate, which was managed with alendronate 70 mg weekly orally. No patient developed osteonecrosis of the jaw.

As the effect of denosumab on the growing skeleton remains unknown, extreme caution was utilized in monitoring for potential skeletal side effects. All four cases were skeletally immature during the denosumab treatment course. Height *z*-score remained stable (Case 1 and 3) or increased (Case 2 and 4). Height *z*-score increase (+1 standard deviation score) in Case 2 was attributed to concurrent treatment with growth hormone. At baseline, BMD assessed by DXA was low in two of four cases, and although within the normal range, below the 50th percentile for the other two cases. BMD increased in all patients but did not exceed the normal range. All four cases experienced extremity/joint pain coinciding with hypercalcemia. One case also reported extremity/joint pain during treatment with denosumab that was most intense in the week following injections and improved within 10 days. X-rays were obtained in three of the four cases and were all notable for metaphyseal sclerosis and widening of the growth plates, similar to the bone changes observed in children treated with bisphosphonates (29) (Supplemental Figure 1). Noonan syndrome is also associated with pigmented villonodular synovitis, a benign proliferative disorder of the large synovial joints or of the tendon sheath of small joints. Presenting symptoms including painless swelling of the joints (30). The resolution of symptoms within a few days of each case did not warrant tissue biopsy to evaluate for this but may be a unique risk factor in the NL/MGCL population.

Osteonecrosis of the jaw (ONJ) has been noted to occur in up to 1% of adults receiving denosumab treatment for giant cell tumors (19), and one case has been described in the literature describing ONJ in a child (25). To reduce risk of ONJ, all patients were counseled on good dental hygiene. They were instructed to continue with routine dental care, including brushing and flossing twice a day, and encouraged to set up regular checkups with their dentist for routine cleanings and necessary care. In this case series, none of the patients developed ONJ, even with the targeted lesions being within the jaw. There is suspicion that the risk of ONJ may be associated with a cumulative dose. In the Uday case, the patient who developed ONJ had an unresectable sacral giant cell tumor that was treated with denosumab, 120 mg loading, then monthly for a total of 46 doses, with a total treatment dose of 5,520 mg. In comparison, cases 1–4 received a total denosumab dose of 460, 1,080, 210, and 390 mg, respectively, which is significantly less. In NL/MGCLs, dosing denosumab using a weight-based dose may help reduce risk of adverse effects while still maintaining efficacy.

We report that a weight-adjusted denosumab dose improved NL/MGCLs. There is limited information in the literature regarding the medical treatment of NL-MGCLs, and there are no randomized clinical trials comparing or showing efficacy of specific medical treatment for NL-MGCLs. In this case series, denosumab treatment has shown excellent results. Alternatively intra-lesional injection of corticosteroids, systemic calcitonin, and interferon has been reported for the treatment of central giant cell granulomas of the jaw; but, they have shown variable results (31, 32). More recently, oral imatinib has been used for treatment of isolated central giant cell lesions and those associated with cherubism (33); however, the data are limited to case reports and small series. The patients with NL/MGCLs in this study have shown excellent response in regression of the tumor with minimal side effects using denosumab. Strengths of the study include usage of a uniform dose among cases for a minimum of 6 months. Limitations of the study include limited sample size, lack of control for comparison, and variation in monitoring for safety and efficacy. Despite the limitations, all four patients demonstrated a significant improvement in NL/MGCLs and were able to avoid further surgery.

Interdisciplinary care, including genetics, oral surgery, and endocrinology, was an important factor in monitoring both safety and efficacy. To avoid hypocalcemia while on denosumab, laboratory data support the optimization of 25-OH vitamin D status prior to and supplementation with calcium if history elicits a calcium deficient diet. At the cessation of denosumab therapy, hypercalcemia occurred within a finite window of 10–11 weeks after the last dose with delay of hypercalcemia noted with preemptive treatment strategies. Based on our collective knowledge gained in these four cases, a proposed treatment regimen for further long-term studies to evaluate safety and efficacy of denosumab in NL/MGCLs would include monthly subcutaneous denosumab 1.7 mg/kg for 6 months. Serum calcium should be monitored periodically throughout treatment and at least 4 months after cessation. Monitoring urine calcium to creatinine ratio may be useful after the cessation of therapy as well as hypercalcemia appears to occur after renal calcium excretion capacity is exceeded. Administration of a bisphosphonate (either orally or intravenously) 8–10 weeks after cessation of denosumab may help prevent rebound hypercalcemia. On-going monitoring of NL/MGCLs continues and is necessary to assess for disease recurrence.

## Data Availability Statement

The raw data supporting the conclusions of this article will be made available by the authors, without undue reservation.

## Ethics Statement

The studies involving human participants were reviewed and approved by Johns Hopkins Institutional Review Board, Baltimore, MD 21205. Written informed consent to participate in this study was provided by the participants' legal guardian/next of kin. Written informed consent was obtained from the minor(s)' legal guardian/next of kin for the publication of any potentially identifiable images or data included in this article.

## Author Contributions

KF, BS, YY, and JLC wrote the manuscript. KF, BS, JC, and JLC collected the data, KF and SK organized and data, and KF, YY, JC, JN, MB, and JLC followed up the case. MB and JLC supervised the management and follow up of the case. All authors revised and approved the final manuscript and agreed to be accountable for the content of the work.

## Conflict of Interest

The authors declare that the research was conducted in the absence of any commercial or financial relationships that could be construed as a potential conflict of interest.
