# Supplementary figures and images for: Case Report: Safety and Efficacy of Denosumab in Four Children With Noonan Syndrome With Multiple Giant Cell Lesions of the Jaw
Source: Front Pediatr. 2020 Sep 18;8:515. doi: 10.3389/fped.2020.00515 (PMC7530181; doi:10.3389/fped.2020.00515)

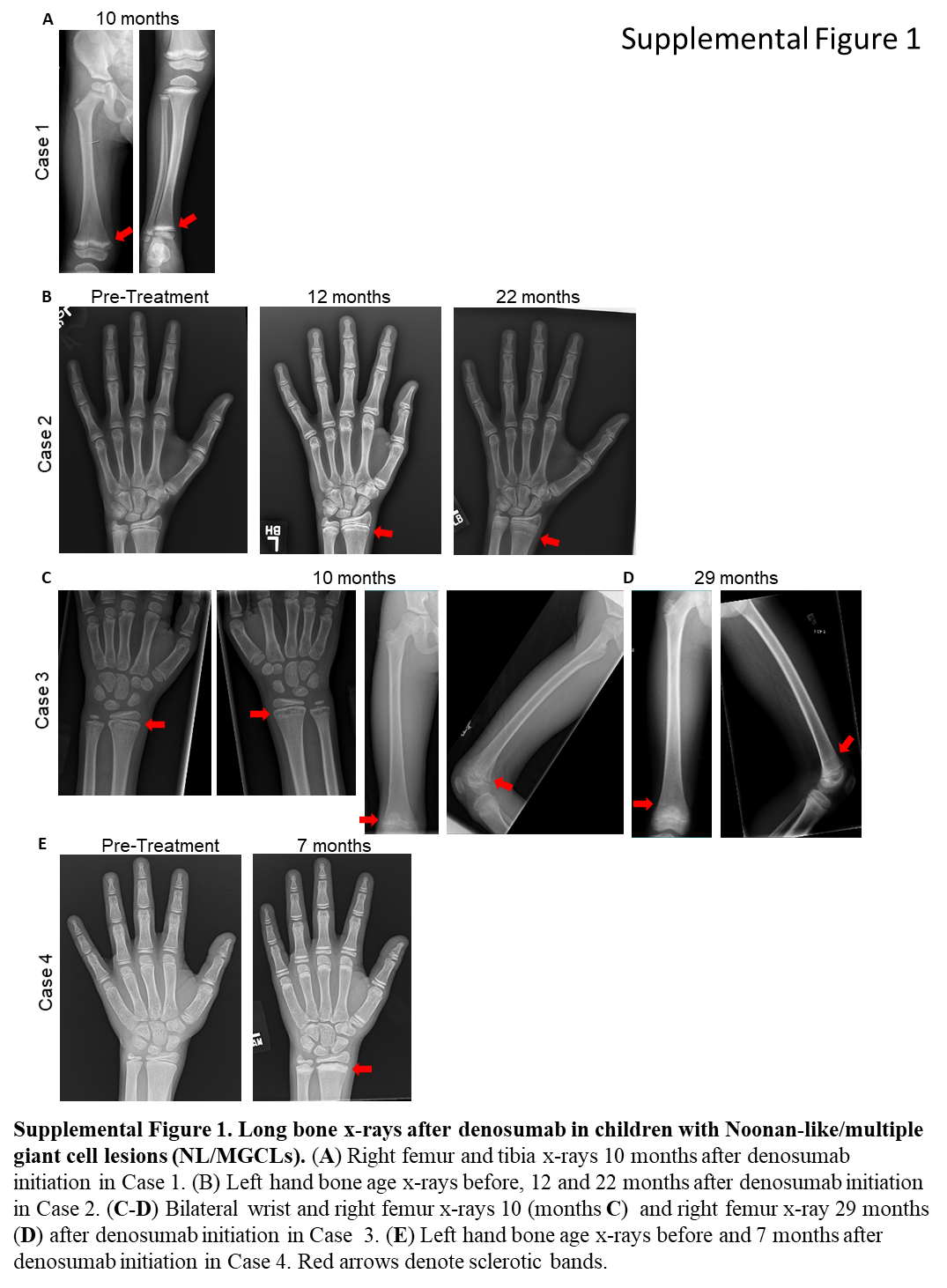

Supplement: Supplementary file 2 [file Image_1.TIFF]
