# Supplementary material for: Case Report: Safety and Efficacy of Denosumab in Four Children With Noonan Syndrome With Multiple Giant Cell Lesions of the Jaw
Source: Front Pediatr. 2020 Sep 18;8:515. doi: 10.3389/fped.2020.00515 (PMC7530181; doi:10.3389/fped.2020.00515)
Supplement: Supplementary file 3 [file Table_1.DOCX]

Supplemental Table 1.

	Additional DXA Sites	Z-score pre-denosumab	Z-score post-denosumab	Change in z-score
Case 1 - 1st treatment round	Right distal femur	-0.2	7.2	7.4
		0.5	0.8	0.3
		-0.4	0.9	1.3
	Left distal femur	-0.3	7.2	7.5
		-0.7	0.8	1.5
		-0.3	0.9	1.2
Case 1 - 2nd treatment round	Right distal femur	-0.2	3.9	4.1
		2.7	0.3	-2.4
		-2.7	-0.1	2.6
	Left distal femur	0.7	3.9	3.2
		-1.2	0.6	1.8
		-2.3	-0.2	2.1
Case 4	Right distal femur	-2.2	-1.0	1.2
		1.2	1.8	0.6
		-2.0	-1.2	0.8
	Left distal femur	1.9	0.5	-1.4
		-1.8	-1.4	0.4
		-2.0	-1.6	0.4
